# Follicle-stimulating hormone associates with prediabetes and diabetes in postmenopausal women

**DOI:** 10.1007/s00592-015-0769-1

**Published:** 2015-05-12

**Authors:** Ningjian Wang, Lin Kuang, Bing Han, Qin Li, Yi Chen, Chunfang Zhu, Yingchao Chen, Fangzhen Xia, Zhen Cang, Chaoxia Zhu, Meng Lu, Ying Meng, Hui Guo, Chi Chen, Dongping Lin, Yingli Lu

**Affiliations:** Institute and Department of Endocrinology and Metabolism, Shanghai Ninth People’s Hospital, Shanghai JiaoTong University School of Medicine, Shanghai, 200011 China; Assisted Reproduction Unit, Department of Obstetrics and Gynecology, Sir Run Run Shaw Hospital, Zhejiang University School of Medicine, Zhejiang, China

**Keywords:** Diabetes, Follicle-stimulating hormone, Postmenopause, Women

## Abstract

**Aims:**

No study explores the association between follicle-stimulating hormone (FSH) and glucose metabolism in general women. We aim to investigate whether the variation of FSH is associated with prediabetes and diabetes in postmenopausal women.

**Methods:**

Our data were from survey on prevalence in East China for metabolic diseases and risk factors in 2014. Thousand six hundred and ten postmenopausal women at the age of 55–89 who were not using hormone replacement therapy were selected. Prediabetes and diabetes were defined according to American Diabetes Association 2014 criteria. FSH, luteinizing hormone, total testosterone and estradiol were measured by chemiluminescence. Multinomial logistic analyses were used for the association of FSH with prediabetes and diabetes, and linear regression for the association of FSH with fasting plasma glucose (FPG) and HbA1c.

**Results:**

Among the participants, 778 (48.3 %) had prediabetes and 121 (7.5 %) had newly diagnosed diabetes. In linear regression, after full adjustment for demographic variables, metabolic factors, E2 and LH, FSH was associated with FPG and HbA1c (*P* < 0.05). In logistic regression, increased quartiles of FSH were associated with significantly decreased odds ratios of prediabetes and diabetes (*P* for trend <0.01). This association was attenuated by waist circumference and HOMA-IR, but persisted in fully adjusted model (*P* for trend <0.05) in which, for the lowest compared with the highest quartile of FSH, the odds ratios of prediabetes and diabetes were 1.93 (95 % CI 1.21–3.08; *P* < 0.01) and 3.02 (95 % CI 1.10–8.31; *P* < 0.05), respectively.

**Conclusions:**

Low FSH was associated with prediabetes and diabetes in postmenopausal women. The associations might be partially explained by adiposity and insulin resistance.

## Introduction

The principal function of sex steroids acts on the reproduction system, but in the recent decade, their roles in the glucose metabolism have also been revealed. In postmenopausal women, endogenous bioavailable testosterone (T) and estradiol (E2) are positively associated with incident type 2 diabetes mellitus (DM) through adiposity and insulin resistance [1, 2].

Follicle-stimulating hormone (FSH) is known as prerequisites for follicular maturation and regulator of ovarian estrogen synthesis in women. However, the role of FSH in glucose metabolism has not been studied. In female dog, FSH plus luteinizing hormone (LH) treatment increases the serum insulin response to glucose load [3]. Increased LH/FSH ratio is a common characteristic of women with polycystic ovary syndrome (PCOS) [4], which is reported to be associated with insulin resistance and obesity in PCOS [5]. A most recent study also found that lower FSH was significantly associated with high prevalence of metabolic syndrome in postmenopausal women, but the sample was relatively small [6]. Though there are no population-based data on the association between FSH and DM in general people, FSH is found to be associated with adiposity in women, which is also a great risk factor for type 2 DM [7–9].

We did a population-based observational investigation named survey on prevalence in East China for metabolic diseases and risk factors (SPECT-China) in 2014 to analyze this association between FSH and type 2 DM in Chinese postmenopausal women older than 55 years. As far as we know, the current analyses are the first one to focus on several possible explanatory factors contributing to the relationship of FSH and type 2 DM, including adiposity, insulin resistance, behavioral and metabolic factors.

## Materials and methods

### Study population

SPECT-China is a cross-sectional survey on prevalence of metabolic diseases and risk factors in East China (ChiCTR-ECS-14005052, www.chictr.org). A stratified and cluster sampling method was used. The first level of sampling was stratified by rural and urban areas and the second level was by economic development area. From February to June 2014, this study was performed in three sites in urban areas of Shanghai, one site in an urban area of Jiangxi Province, three sites in rural areas in Shanghai, three sites in rural areas in Zhejiang and six sites in rural areas in Jiangxi Province. Adults aged 18 years and older who were Chinese citizens and had lived at their current residence for 6 months or longer were invited to participate in our study. Those with severe communication problems, with acute illness or who were unwilling to participate were excluded from the study.

**Fig. 1:**
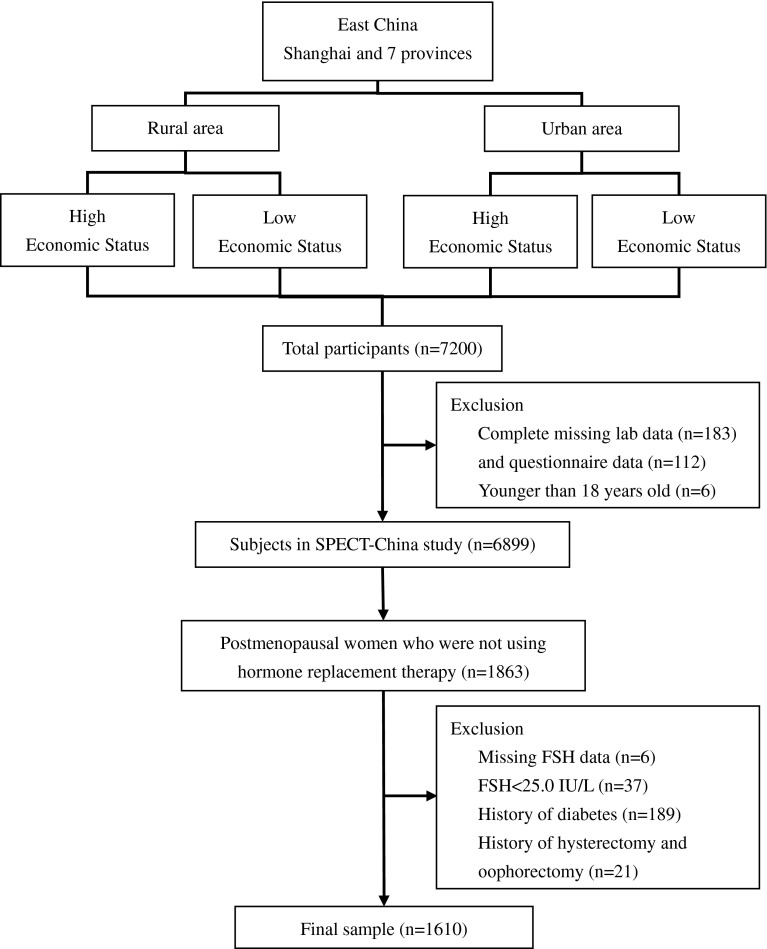
Flowchart of sampling frame and participants selected from SPECT-China. *FPG* fasting plasma glucose

A total of 7200 people participated in this investigation. After exclusion of participants who had missing laboratory results (*n* = 183), missing questionnaire data (*n* = 112) and were younger than 18-year-old (*n* = 6), six thousand eight hundred and ninety-nine subjects were enrolled in SPECT-China study. A woman was considered postmenopausal if she was more than 55 years of age [1, 2, 8, 10]. There were 1863 women who were postmenopausal and were not using hormone replacement therapy. Women with diabetes history (*n* = 189), with FSH <25.0 IU/L (*n* = 37), with missing values of FSH (*n* = 6) and with a history of hysterectomy and oophorectomy (*n* = 21) were excluded. Finally, this study was based on a total number of 1610 postmenopausal women (Fig. 1).

The study protocol was approved by the Ethics Committee of Shanghai Ninth People’s Hospital, Shanghai JiaoTong University School of Medicine. All procedures followed were in accordance with the ethical standards of the responsible committee on human experimentation (institutional and national) and with the Helsinki Declaration of 1975, as revised in 2008. Informed consent was obtained from all patients for being included in the study.

### Biochemical measurements

Venous blood samples were drawn after an overnight fast of at least 8 h. The blood samples for plasma glucose test were collected into vacuum tubes with anticoagulant sodium fluoride and centrifuged on the spot in 1 h after collection. Blood samples were stored at −20 °C when collected and shipped by air in dry ice to a central laboratory within 2–4 h of collection, which was certified by the College of American Pathologists. Glycated hemoglobin (HbA1c) was assessed by high-performance liquid chromatography (MQ-2000PT, China). Plasma glucose and lipid profile including total cholesterol, triglycerides, high-density lipoprotein (HDL) and low-density lipoprotein (LDL) were measured by BECKMAN COULTER AU 680 (Germany). Insulin was detected by chemiluminescence method (Abbott i2000 SR, USA).

Total T, E2, FSH and LH were measured by chemiluminescence (SIEMENS Immulite 2000, Germany). The minimal detectable limit for each hormone was as follows: 0.7 nmol/L (total T), 73.4 pmol/L (E2) and 0.1 IU/L (FSH and LH). The inter-assay coefficients of variation were 6.6 % (total T), 7.5 % (E2), 4.5 % (FSH) and 6.0 % (LH). The intra-assay coefficients of variation were 5.7 % (total T), 6.2 % (E2), 3.8 % (FSH) and 4.9 % (LH).

### Clinical and anthropometric measurements

In every site, the same staff group collected all the data. They were trained according to a standard protocol that made them familiar with the specific tools and methods used. Trained staff used a questionnaire to collect information on demographic characteristics, medical history and lifestyle risk factors. Current smoking was defined as having smoked at least 100 cigarettes in one’s lifetime and currently smoking cigarettes [11]. Self-reported educational levels from illiteracy, junior and senior high school, college to postgraduate were recorded. We classified them into illiteracy and non-illiteracy. Body weight, height, waist circumference and blood pressure were measured with the use of standard methods as described previously [11]. Body mass index (BMI) was calculated as weight in kilograms divided by height in meters squared. Insulin resistance was estimated by the homeostatic model assessment (HOMA-IR) index: [fasting insulin (mIU/L)] × [FPG (mmol/L)]/22.5.

### Definition of variables

In accordance with American Diabetes Association 2014 criteria, prediabetes was defined as impaired fasting glucose [fasting plasma glucose (FPG) 5.6–6.9 mmol/L] or HbA1c concentrations between 5.7 and 6.4 %, or both, while diabetes was defined as a previous diagnosis by healthcare professionals, FPG 7.0 mmol/L or higher or HbA1c 6.5 % or higher.

In China, the prevalence of diabetes in rural and urban areas is different [11]. Therefore, we took residence area as a covariate. Economic development status also affects diabetes prevalence [11]. Current economic status was assessed by gross domestic product (GDP) per capita of 2013 in each study site. The mean national GDP per capita (6807 US dollars from World Bank) in 2013 was considered as the cutoff point for economic status.

### Statistical analysis

We performed survey analyses with IBM SPSS Statistics, Version 22 (IBM Corporation, Armonk, NY, USA). All analyses were two-sided. A *P* value <0.05 was taken to indicate a significant difference. General demographic and laboratory characteristics are summarized as median with interquartile range (IQR) for continuous variables or as number with proportion for categorical variables. To test for differences of characteristics among different glucose tolerance status and FSH quartiles, Kruskal–Wallis test was used for continuous data with skewed distribution, and Pearson chi-squared test was used for categorical variables. A part of total T (67.7 %) and E2 (66.3 %) was under the minimal detectable limit, and samples with values below the minimal detectable limit were given a value midway between zero and the minimal detectable limit for the analyses: 0.35 nmol/L for total T and 36.7 pmol/L for E2 (10).

The association of FSH (independent variable) with FPG and HbA1c (dependent variables) was assessed by linear regression. Model 1 included terms for age, residence area, economic status and LH. Model 2 included terms for model 1 and E2. Model 3 included terms for model 2, waist circumference and HOMA-IR. Since waist circumference and BMI were highly correlated (Spearman’s correlation coefficient = 0.72; *P* < 0.01), only waist circumference was used as a measure of adiposity. Model 4 was a fully adjusted model including all covariates in model 3, LDL, HDL, triglycerides, systolic blood pressure and current smoker. Since FPG and HbA1c were non-normally distributed, they were log-transformed. Results were expressed as standardized coefficients. *R*^2^ represented the coefficient of determination.

FSH and LH were divided into quartiles, with the first quartile representing the lowest one and the fourth quartile the highest. Odds ratio (OR) and 95 % confidence intervals (CI) were calculated using multinomial logistic regression to determine the risk of diabetes and prediabetes for each quartile of FSH and LH, using the highest quartile as the reference. Besides models in linear regression, we also adjusted models for waist circumference and HOMA-IR separately. Interaction effect was tested between FSH and residence area, economic status and waist circumference by adding a multiplicative factor in the logistic regression model.

Sensitivity analyses were performed by additional adjustment for total T, substituting BMI for waist circumference in multivariable models. We also conducted further sensitivity analyses excluding cases whose E2 higher than minimal detectable limit (73.4 pmol/L). Because our menopause was based on age, we performed the regression analyses in women older than 60 years.

## Results

### Characteristics of the study population

General demographic and laboratory characteristics of the study population are shown in Table 1. This study recruited 1610 postmenopausal women. Among them, 711 (44.2 %) had normal glucose regulation (NGR), 778 (48.3 %) had prediabetes, and 121 (7.5 %) had newly diagnosed diabetes. Compared with postmenopausal women with NGR, women with diabetes were significantly older and more likely to be residents in rural and high economic development area. These women also had significantly greater BMI, waist circumference, fasting insulin, HOMA-IR, triglycerides and systolic pressure. Compared with the participants with NGR, women with prediabetes and diabetes had comparable levels of total T and E2, but lower levels of FSH [62.4 (31.4) and 54.9 (28.4) vs 69.3 (32.9) IU/L, *P* < 0.01].

**Table 1:** General characteristics of postmenopausal women by glycemic status

	NGR	Prediabetes	Newly diagnosed diabetes	*P*
*N*	711	778	121	
Age (year) [min, max]	62 (8) [55, 84]	63 (9) [55, 89]	64 (8) [55, 89]	<0.05
Metabolic factors				
BMI (kg/m^2^)	23.8 (4.1)	24.2 (4.8)	25.9 (4.4)	<0.01
Wait circumference (cm)	78.0 (12.0)	80.0 (13.8)	82.0 (13.0)	<0.01
HbA1c (%)	5.2 (0.5)	5.6 (0.6)	6.2 (1.1)	<0.01
Fasting glucose (mmol/L)	5.14 (0.47)	5.90 (0.56)	7.50 (1.18)	<0.01
Fasting insulin (pmol/L)	31.0 (19.2)	35.0 (23.3)	51.4 (54.5)	<0.01
HOMA-IR	1.01 (0.65)	1.30 (0.94)	2.45 (2.77)	<0.01
LDL-cholesterol (mmol/L)	3.06 (0.95)	3.04 (0.96)	3.10 (1.04)	0.31
HDL-cholesterol (mmol/L)	1.55 (0.43)	1.49 (0.38)	1.43 (0.49)	<0.01
Triglycerides (mmol/L)	1.32 (0.82)	1.40 (0.86)	1.77 (1.36)	<0.01
Systolic pressure (mmHg)	134.0 (27.8)	136.0 (28.0)	147.0 (30.0)	<0.01
Sex-related hormones				
Total T (nmol/L)	0.35 (0.45)	0.35 (0.45)	0.35 (0.65)	0.37
E2 (pmol/L)	36.7 (53.0)	36.7 (52.4)	36.7 (62.4)	0.82
FSH (IU/L)	69.3 (32.9)	62.4 (31.4)	54.9 (28.4)	<0.01
LH (IU/L)	24.4 (12.8)	22.9 (12.7)	21.0 (13.2)	<0.01
Demographics				
Illiteracy (%)	28.4	29.2	28.6	0.95
Current smoker (%)	2.9	4.4	5.5	0.20
Residence area {rural/urban (%)}	63.0/37.0	81.6/18.4	79.3/20.7	<0.01
Economic status {low/high (%)}	29.0/71.0	22.2/77.8	14.9/85.1	<0.01

Data were summarized as median (interquartile range) for continuous variables or as number with proportion for categorical variables. Kruskal–Wallis test was used for continuous variables with skewed distribution and Pearson chi-squared test for dichotomous variables

*NGR* normal glucose regulation, *HOMA*-*IR* homeostasis model assessment-insulin resistance, *T* testosterone, *E2* estradiol, *BMI* body mass index, *FSH* follicle-stimulating hormone, *LH* luteinizing hormone, *HbA1c* glycated hemoglobin, *HDL* high-density lipoprotein, *LDL* low-density lipoprotein

Characteristics of postmenopausal women according to serum FSH quartiles are summarized in Table 2. The quartile ranges of FSH in postmenopausal women were ≤50.2, 50.3–64.8, 64.9–82.4 and ≥82.5 IU/L. Compared with women in the highest quartile, women in the lowest quartile had comparable ages, but greater BMI, waist circumference, HbA1c, fasting glucose, fasting insulin, HOMA-IR, triglycerides and systolic pressure. They also had similar total T level, but significantly higher E2.

**Table 2:** Characteristics of postmenopausal women according to serum follicle-stimulating hormone quartiles

	Q1	Q2	Q3	Q4	*P*
*N*	406	400	403	401	
FSH (IU/L)	≤50.2	50.3–64.8	64.9–82.4	≥82.5	
Age (year)	63 (9)	63 (9)	62 (9)	62 (9)	0.19
Metabolic factors					
BMI (kg/m^2^)	25.2 (4.9)	24.4 (4.6)	23.8 (4.4)	23.5 (3.9)	<0.01
Wait circumference (cm)	82.0 (15.0)	80.0 (12.0)	78.0 (13.0)	77.0 (13.0)	<0.01
HbA1c (%)	5.5 (0.6)	5.4 (0.6)	5.3 (0.6)	5.3 (0.6)	<0.01
Fasting glucose (mmol/L)	5.66 (1.00)	5.56 (0.88)	5.50 (0.89)	5.39 (0.81)	<0.01
Fasting insulin (pmol/L)	36.6 (26.9)	33.9 (23.6)	33.9 (21.9)	30.9 (19.1)	<0.01
HOMA-IR	1.32 (1.16)	1.20 (0.90)	1.20 (0.82)	1.10 (0.75)	<0.01
LDL-cholesterol (mmol/L)	3.07 (0.97)	3.07 (1.03)	2.98 (0.95)	3.09 (0.88)	0.26
HDL-cholesterol (mmol/L)	1.48 (0.39)	1.49 (0.44)	1.52 (0.40)	1.58 (0.45)	<0.01
Triglycerides (mmol/L)	1.47 (0.97)	1.43 (1.03)	1.36 (0.88)	1.32 (0.79)	<0.05
Systolic pressure (mmHg)	138.0 (29.0)	136.0 (26.0)	135.0 (29.0)	134.0 (28.3)	<0.05
Sex-related hormones					
Total T (nmol/L)	0.35 (0.48)	0.35 (0.55)	0.35 (0.45)	0.35 (0.45)	0.27
E2 (pmol/L)	36.7 (77.3)	36.7 (56.8)	36.7 (41.3)	36.7 (0)	<0.01
LH (IU/L)	15.7 (6.9)	20.8 (7.4)	25.3 (8.0)	34.2 (11.8)	<0.01
Demographics					
Illiteracy (%)	31.5	29.4	24.7	29.8	0.25
Current smoker (%)	3.8	3.0	5.4	2.7	0.23
Residence area {rural/urban (%)}	76.8/23.2	73.3/26.8	71.5/28.5	71.3/28.7	0.25
Economic status {low/high (%)}	25.4/74.6	28.0/72.0	23.8/76.2	21.4/78.6	0.18

Data were summarized as median with interquartile range for continuous variables or as number with proportion for categorical variables. Kruskal–Wallis test was used for continuous variables with skewed distribution and Pearson chi-squared test for dichotomous variables

*NGR* normal glucose regulation, *HOMA*-*IR* homeostasis model assessment-insulin resistance, *T* testosterone, *E2* estradiol, *BMI* body mass index, *FSH* follicle-stimulating hormone, *LH* luteinizing hormone, *HbA1c* glycated hemoglobin, *HDL* high-density lipoprotein, *LDL* low-density lipoprotein

### Association of FSH with FPG and HbA1c

Table 3 summarizes the results of the linear regression models studying the association of FSH with FPG and HbA1c. In base model (Table 3, model 1), higher FSH levels were associated with lower log FPG (standardized *β* = −0.138) and log HbA1c (standardized *β* = −0.138; both *P* < 0.001). Further adjustment for E2 did not obviously attenuate the association and change *R*^2^ (Table 3, model 2). After further adjustment for waist circumference and HOMA-IR, this association largely weakened and *R*^2^ changed greatly from 0.11 to 0.31 for log FPG and from 0.03 to 0.06 for log HbA1c, but there was still statistical significance (Table 3, model 3). Further adjustment for LDL, HDL, triglycerides and systolic blood pressure and current smoker did not change the association, and there was no change in *R*^2^ for log FPG (Table 3, model 4).

**Table 3:** Association of FSH with FPG and HbA1c: linear regression

Dependent variables	Standardized *β*	*P* value	*R* ^2^
Log FPG (model 1)	−0.138	<0.001	0.11
Log FPG (model 2)	−0.152	<0.001	0.11
Log FPG (model 3)	−0.092	0.007	0.31
Log FPG (model 4)	−0.087	0.011	0.31
Log HbA1c (model 1)	−0.138	<0.001	0.03
Log HbA1c (model 2)	−0.135	0.001	0.03
Log HbA1c (model 3)	−0.091	0.022	0.06
Log HbA1c (model 4)	−0.097	0.014	0.09

*R* ^2^ represented the coefficient of determination

Since FPG and HbA1c were non-normally distributed, they were log-transformed

Model 1 included terms for age, residence area, economic development and luteinizing hormone

Model 2 included terms for model 1 and E2

Model 3 included terms for model 2, waist circumference and HOMA-IR

Model 4 was a fully adjusted model including all covariates in model 3, metabolic factors [low-density lipoprotein, high-density lipoprotein, triglycerides and systolic blood pressure] and current smoker

*FPG* fasting plasma glucose, *HbA1c* glycated hemoglobin

### Association of FSH with prediabetes and diabetes

Multinomial logistic regression analyses (Table 4) showed that the risk of prevalent prediabetes and newly diagnosed diabetes decreased across FSH quartiles (*P* for trend <0.05 in every model). Compared with women in the highest quartile of FSH (Table 4, model 1), ORs of prediabetes and diabetes in women in the lowest quartile of FSH were 1.96 (95 % CI 1.30, 2.93; *P* < 0.001) and 4.68 (95 % CI 2.02, 10.82; *P* < 0.01), respectively. Adjustment for E2 did not weaken the association of FSH with prediabetes and diabetes (Table 4, model 2).

**Table 4:** Association of circulating follicle-stimulating hormone and luteinizing hormone with prediabetes and diabetes in postmenopausal women

	Model 1	Model 2	Model 3	Model 4	Model 5	Model 6
Prediabetes						
FSH (IU/L)						
Q1 (≤50.2)	1.96 (1.30, 2.93)^†^	2.12 (1.40, 3.20)^#^	1.99 (1.29, 3.08)^†^	1.81 (1.18, 2.79)^†^	1.79 (1.14, 2.79)*	1.93 (1.21, 3.08)^†^
Q2 (50.3–64.8)	1.58 (1.11, 2.25)*	1.64 (1.15, 2.33)^†^	1.48 (1.03, 2.14)*	1.49 (1.03, 2.14)*	1.40 (0.96, 2.04)	1.49 (1.00, 2.20)*
Q3 (64.9–82.4)	1.18 (0.86, 1.62)	1.20 (0.87, 1.65)	1.24 (0.89, 1.72)	1.14 (0.82, 1.58)	1.19 (0.85, 1.66)	1.23 (0.87, 1.75)
Q4 (≥82.5)	1.00	1.00	1.00	1.00	1.00	1.00
*P* value for trend	<0.001	<0.001	0.002	0.003	0.009	0.004
LH (IU/L)						
Q1 (≤17.9)	0.81 (0.54, 1.22)	0.80 (0.53, 1.20)	0.79 (0.52, 1.21)	0.82 (0.54, 1.25)	0.86 (0.55, 1.33)	0.88 (0.56, 1.39)
Q2 (18.0–23.6)	0.91 (0.64, 1.28)	0.89 (0.63, 1.26)	0.90 (0.62, 1.29)	0.88 (0.62, 1.26)	0.95 (0.65, 1.37)	0.91 (0.62, 1.34)
Q3 (23.7–30.7)	0.84 (0.62, 1.15)	0.83 (0.61, 1.14)	0.82 (0.59, 1.13)	0.83 (0.60, 1.15)	0.83 (0.59, 1.15)	0.83 (0.59, 1.17)
Q4 (≥30.8)	1.00	1.00	1.00	1.00	1.00	1.00
*P* value for trend	0.38	0.34	0.35	0.41	0.62	0.65
Diabetes						
FSH (IU/L)						
Q1 (≤50.2)	4.68 (2.02, 10.82)^#^	5.14 (2.19, 12.05)^#^	3.60 (1.48, 8.75)^†^	3.59 (1.43, 9.02)^†^	2.75 (1.06, 7.17)*	3.02 (1.10, 8.31)*
Q2 (50.3–64.8)	3.64 (1.67, 7.93)^†^	3.80 (1.74, 8.32)^†^	2.93 (1.31, 6.53)^†^	3.03 (1.30, 7.07)*	2.48 (1.05, 5.89)*	2.43 (0.97, 6.13)
Q3 (64.9–82.4)	2.78 (1.34, 5.77)^†^	2.82 (1.36, 5.87)^†^	2.38 (1.12, 5.04)*	2.15 (0.97, 4.76)	1.62 (0.71, 3.69)	1.76 (0.74, 4.16)
Q4 (≥82.5)	1.00	1.00	1.00	1.00	1.00	1.00
*P* value for trend	0.001	<0.001	0.007	0.007	0.029	0.030
LH (IU/L)						
Q1 (≤17.9)	1.13 (0.54, 2.37)	1.11 (0.53, 2.33)	1.03 (0.47, 2.26)	1.18 (0.51, 2.69)	1.15 (0.48, 2.76)	1.10 (0.44, 2.79)
Q2 (18.0–23.6)	0.72 (0.35, 1.45)	0.70 (0.35, 1.43)	0.67 (0.32, 1.41)	0.67 (0.30, 1.47)	0.73 (0.32, 1.68)	0.67 (0.28, 1.61)
Q3 (23.7–30.7)	0.89 (0.46, 1.71)	0.88 (0.46, 1.69)	0.82 (0.41, 1.62)	0.94 (0.46, 1.94)	0.86 (0.40, 1.83)	0.77 (0.35, 1.72)
Q4 (≥30.8)	1.00	1.00	1.00	1.00	1.00	1.00
*P* value for trend	0.70	0.75	0.87	0.75	0.72	0.76

Model 1 included terms for age, residence area and economic status

Model 2 included terms for model 1 and E2

Model 3 included terms for model 2, waist circumference

Model 4 included terms for model 2, HOMA-IR

Model 5 included terms for model 2, waist circumference and HOMA-IR

Model 6 was a fully adjusted model including all covariates in model 5, metabolic factors [waist circumference, HOMA-IR, low-density lipoprotein, high-density lipoprotein, triglycerides and systolic blood pressure] and current smoker

No interaction was found between FSH and residence area, economic status and waist circumference

Data were odds ratio (95 % CI). * *P* < 0.05; ^†^ *P* < 0.01; ^#^ *P* < 0.001

After further adjustment for waist circumference based on model 2, the *P* value changed from <0.001 to 0.002 in prediabetes and from <0.001 to 0.007 in diabetes (Table 4, model 3). Based on model 2, further adjustment for HOMA-IR changed the *P* value from <0.001 to 0.003 in prediabetes and from <0.001 to 0.007 in diabetes (Table 4, model 4). Thus, waist circumference and HOMA-IR comparably attenuated the association between FSH and diabetes. Adjusting for both waist circumference and HOMA-IR weakened the association between FSH and diabetes such further that it was no longer significant in Q3 [OR = 1.62 (95 % CI 0.71, 3.69), *P* > 0.05] (Table 4, model 5). Further adjustment for LDL, HDL, triglycerides and systolic blood pressure attenuated this association further in Q2 [OR = 2.43 (95 % CI 0.97, 6.13), *P* > 0.05] (Table 4, model 6), but in Q1, there was still statistical significance [OR = 3.02 (95 % CI 1.10, 8.31), *P* < 0.05]. It was worth mentioning that LH did not show association with diabetes and prediabetes in every model. No interaction was found between FSH and residence area, economic status and waist circumference.

### Sensitivity analysis

In sensitivity analysis, using BMI instead of waist circumference in relevant models did not change the observed association (both *P* for trend <0.05). Additional adjustment for total testosterone also did not alter the association (both *P* for trend <0.05). Furthermore, after exclusion of cases whose E2 was higher 73.4 pmol/L, the association of FSH with prediabetes and diabetes did not significantly change in fully adjusted model (both *P* for trend <0.05). Even we raised the cutoff age of menopause to 60 years, the significant association still exists (both *P* for trend <0.05).

## Discussion

In this study, we found that higher FSH level was significantly associated with lower FPG and HbA1c and with lower risk of prevalent prediabetes and diabetes in postmenopausal women. Adiposity and insulin resistance may partially explain this association. As far as we know, this is the first study to detect the association between FSH level and prediabetes and diabetes in a population-based investigation with a large sample.

Previously, the association of FSH with metabolic disorders was mainly described in premenopausal women with polycystic ovary syndrome (PCOS). Low-normal FSH level, increased serum LH level, and increased LH/FSH ratio have been recognized as common characteristics of women with PCOS [4]. LH/FSH ratio more than 2.5 is believed to be useful to identify women with PCOS [6]. Some reported that it is associated with insulin resistance and obesity in PCOS [5], but another study showed an inconsequential predictive value of the LH/FSH ratio on insulin resistance, which needs further study [12].

In our study, the diabetic patients were older than normal subjects. And in a previous study, it was observed that concentration of FSH declined with aging in women over 70 years [13]. Some may be wondering whether the association between FSH and diabetes was actually because of aging. However, in Table 2, among the FSH quartiles, the age did not significantly decreased or increased (*P* = 0.19). According to correlation analyses, age was not significantly correlated with FSH in our subjects (Spearman’s correlation coefficient = −0.04, *P* = 0.092). Meanwhile, in regression models, age was also adjusted. Therefore, we think aging may not affect the association between FSH and diabetes in our study.

We observed that FSH was associated with diabetes partially through its relation to waist circumference and insulin resistance. In Chinese adults, waist circumference may be better than BMI as an alternative measure of body fatness or fat distribution for predicting diabetic and cardiovascular risks [14, 15], and waist circumference and BMI were highly correlated (Spearman’s correlation coefficient = 0.72; *P* < 0.01), so we chose waist circumference for adjustment instead of BMI. Previous studies found that FSH was lower in obese participants [7, 16] and that weight loss could even elevate FSH level in overweight postmenopausal women [8]. Some explained that low FSH in obesity could be attributed to increased production of endogenous estrogens by mesenchymal adipose tissue [17]. Cross-sectional and prospective studies found significant relationship between E2 and diabetes [1, 2] and insulin resistance, independent of adiposity [1]. In our study, however, E2 was comparable in NGR, prediabetes and diabetes, despite significantly different values of FSH, and adjustment for E2 did not attenuate the association between FSH and diabetes (Table 4, model 2). There may be other types of estrogens playing their roles in this association. Obese women tend to have higher free E2, higher estrone and lower SHBG [6]. It is reasonable that high free E2 could suppress FSH. High free E2 and lower SHBG were reported to significantly increase risk of developing diabetes in postmenopausal women [18]. Moreover, more significant correlations in estrone and BMI than in E2 have been observed [19]. It is worth mentioning that in our study after adjustment for adiposity and other metabolic factors, FSH still significantly associated with prediabetes and diabetes. Therefore, other underlying mechanisms should be further explored.

We also speculate that FSH may associate with diabetes through inflammatory markers. Low-grade systemic inflammation was related to development of diabetes [20]. Inflammatory markers, such as C-reactive protein, TNF-α and IL-1β, not only were positively associated with estrone level [21], but also could suppress gonadotropin-releasing hormone release in animal studies [22], which is also common in diabetic men [23]. The unexplained part of association between FSH and diabetes in our study may be due to inflammatory markers, which needs further study.

Two recent studies proved that FSH was a biomarker to assess the probability of metabolic syndrome better than C-reactive protein, leptin or SHBG in postmenopausal women [6, 24]. Stefanska et al.’s [24] study indicated that the association between FSH and metabolic syndrome is mainly explained by obesity but not by an association with E2, which is consistent with our results. However, the pathophysiology of the relationship between FSH, adiposity and diabetes is not well determined; in our study, we speculate that FSH may be a protective biomarker of glucose metabolism in postmenopausal women.

The study had some strengths. First, the novelty, it is the first study to detect the association between FSH level and glucose metabolism in a large population-based sample. Second, anthropometric measurements and questionnaires were completed by the same trained research group with strong quality control. Third, our data source is SPECT-China study that was performed in a general population as opposed to a clinic-based population, so the results may be more reflective. However, our study also has some limitations. First, because of cross-sectional study nature, we cannot draw causal relationship between FSH and diabetes. Second, though self-reported age at menopause is the clearest way to classify menopausal status [25], we considered women older than 55 years could be postmenopausal. In China, the overall median age at natural menopause is 50 years, and at the age of 55 years, 97 % of women are postmenopausal [26]. Even we raised the cutoff age to 60, the association between FSH and diabetes did not change in fully adjusted model. Thus, we do not expect that this would seriously bias this study. Third, we only measured FSH and E2 for a single time. However, this may not largely affect the results because FSH and E2 are considered to be stable about 2 years after final menstrual period [16]. Finally, we could not collect PCOS data. The first report about PCOS in China we could find was published in 1989 [27], and first Chinese diagnostic criteria were established in 2012. Therefore, when our subjects were at reproductive age, PCOS was not well recognized by physicians and patients two decades ago. Participants may not provide correct information about PCOS. Moreover, the elevation of LH concentrations is the main biochemical abnormality of PCOS [28], but our study focused on the FSH.

In conclusion, low FSH was associated with higher FPG and HbA1c and also with higher prevalence of prediabetes and diabetes in postmenopausal women. These associations might be partially explained by adiposity and insulin resistance. Whether FSH is a protective biomarker of glucose metabolism in postmenopausal women needs further exploration.
